# The Potential for Genotoxicity, Mutagenicity and Endocrine Disruption in Triclosan and Triclocarban Assessed through a Combination of In Vitro Methods

**DOI:** 10.3390/jox14010002

**Published:** 2023-12-21

**Authors:** Jan Chrz, Markéta Dvořáková, Kristina Kejlová, Danuše Očadlíková, Lada Svobodová, Lukáš Malina, Barbora Hošíková, Dagmar Jírová, Hana Bendová, Hana Kolářová

**Affiliations:** 1 Centre of Toxicology and Health Safety, National Institute of Public Health, Šrobárova 49/48, 100 00 Prague, Czech Republic; 2 Department of Medical Biophysics, Institute of Molecular and Translational Medicine, Faculty of Medicine and Dentistry, Palacky University in Olomouc, 779 00 Olomouc, Czech Republic

**Keywords:** genotoxicity/mutagenicity, preservatives, chromosome aberrations, Ames test, Comet assay, endocrine disruption

## Abstract

Triclosan and Triclocarban, preservatives widely used in cosmetics and other consumer products, underwent evaluation using a battery of new-approach methodologies in vitro (NAMs). Specifically, the Microplate Ames Test (MPF™ Test, Xenometrix, Allschwil, Switzerland) was employed to assess mutagenicity, the Comet assay in vitro on the HaCat cell line and the Mammalian Chromosome Aberration Test were utilized to evaluate genotoxicity, and the XenoScreen^®^ YES/YAS assay was applied to investigate endocrine disruption. The chemicals did not exhibit any positive responses for mutagenicity. However, the mammalian chromosome aberration test identified both chemicals as being positive for genotoxicity at 10 µg/mL. In the Comet assay, the percentage of DNA in the tail significantly increased in a concentration-dependent manner (at 5 and 10 µg/mL for Triclosan, at 2.5, 5, and 10 µg/mL for Triclocarban). The positive response depended on the increasing concentration and the duration of exposure. Triclosan, but not Triclocarban in any of the endocrine assays performed, indicated a potential for endocrine activity in the anti-estrogenic and anti-androgenic assays. The positive in vitro results detected were obtained for concentrations relevant to final products. The alarming findings obtained with the use of new-approach methodologies (NAMs) justify the current precautionary regulatory approach, limiting the use of these preservatives.

## 1. Introduction

Triclosan (TCS) and Triclocarban (TCC) have been used for decades, since the early 1940s, as fungicides and preservatives in various products, including clothing, fabric and leather finishing agents, toys, food packaging, floors in the food industry, construction materials, medical supplies and particularly in household and personal care products, such as soaps, antibacterial bar/liquid soaps, body lotions, deodorants, detergents, medical disinfectants, aftershave soaps, hand sanitizers, toothpastes, handwashes and mouthwashes, body washes, cleansing lotions, baby teethers and wipes, detergents, etc. [1]. The two chemicals differ in their structures, but both are polychlorinated aromatic antimicrobials, in which three hydrogen atoms on aromatic rings are substituted with chlorine. They are effective against many different bacteria, as well as some fungi and protozoa, as they inhibit fatty acid synthesis and induce the disruption of membrane integrity [2,3].

Dermal application of personal care products is believed to be the main route of human exposure, although they may also enter the human body orally through toothpaste, mouthwashes and dental treatments. As a result, TCS and TCC have been detected in human samples such as blood [4,5,6], breast milk [7,8], urine [9,10,11,12], hair and nails [13,14]. Moreover, TCS and TCC represent a burden for the aquatic environment and pose a risk of contaminating drinking water, as current wastewater treatment plants are not typically designed for the treatment of such micropollutants [15,16]. The long-term exposure of aquatic organisms to TCS and TCC, coupled with their bioaccumulation potential, has led to detectable levels of them throughout aquatic food chains in species such as algae, crustaceans, fish and marine mammals [17,18]. Environmental TCS and TCC could be efficiently taken up by food crops, leading to additional potential human exposure through food consumption [19,20,21]. Their adverse biological effects in humans, including endocrine disruption, developmental and reproductive impacts [6,22,23,24,25,26], gut microbiota disturbance [21,27,28] and the elevation of antibiotic resistance [29,30,31,32], have led to strict regulations on the usage of both preservatives.

In the EU, according to the current regulation, (EC) No. 1223/2009 [33], on cosmetic products (Cosmetics Regulation), TCS is allowed to be used as a preservative in cosmetic products with a maximum concentration of 0.3% in toothpaste, hand soaps, body soaps/shower gels, deodorants (not sprays), face powders, blemish concealers and nail cleansing products prior to the application of artificial nails. Additionally, it is allowed in mouthwashes with a maximum concentration of 0.2% (entry 25 Annex V). The use of TCC as a preservative is regulated to a maximum concentration of 0.2% in the final product (entry 23 Annex V), and to a maximum concentration of 1.5% in rinse-off products, excluding its use as an inhibitor of microorganisms (entry 100, Annex III). Concerning the potential endocrine disrupting properties of these two preservatives, the Scientific Committee on Consumer Safety has issued recommendations for the currently discussed amendment to the Cosmetic Regulation. It suggests that TCS should not be used in mouthwash or toothpaste for children under 3 years of age, and TCC should not be allowed for use in mouthwash and toothpaste for children under 6 years of age. Labelling requirements should also be introduced with the aim of enhancing the protection of consumers [2].

In 2016, the U.S. Food and Drug Administration (FDA) banned the use of 19 antimicrobial agents, including TCC and TCS, in personal care products, due to their disputable contribution to preventing bacterial diseases, their potential role in the development of resistance in microorganisms, their ecotoxicity and the potential health risks for humans. The FDA concluded that the total available data regarding the safety profile of TCS and TCC do not contain sufficient information to determine whether these preservatives are generally recognized as safe for use in consumer antiseptic wash products [34].

In 2019, the European Commission invited interested parties, including academic and other research institutes, EU countries’ authorities, manufacturers of cosmetic products, producers of substances of concern and consumers associations, to submit any scientific information relevant to the safety assessment of 14 substances, including TCS and TCC [2]. The submission of any relevant scientific information, including the endocrine-disrupting potential of ingredients used in cosmetic products, was required in the framework of the Cosmetics Regulation and the amendment of its annexes listing ingredients that are banned, approved or only authorized for limited use.

Animal testing is prohibited for cosmetic ingredients and final products in the EU by the Cosmetics Regulation. Testing chemical substances of toxicological concern in human volunteers for toxicity endpoints, such as genotoxicity, mutagenicity, carcinogenicity and endocrine disruption, is ethically unacceptable. The use of in vitro toxicological methods remains the only available approach to assess the hazard and risks associated with the use of chemical preservatives present in cosmetic products. Suitable combinations of new-approach methodologies (NAMs) and test systems of human origin (mainly with regard to human genes, receptors, proteins, cells and tissues) providing mechanistic data of the highest human relevance, have recently been suggested as a scientifically valid approach and are currently utilized as efficient non-animal tools for the assessment of genotoxicity and endocrine disruption (OECD Test Guidelines, http://www.oecd-ilibrary.org/ accessed on 15 December 2023). Bacterial strains have been broadly used to predict the potential of mutagenicity. In the Ames test, the assay is usually performed with bacterial strains of *Salmonella typhimurium* or *Escherichia coli*, both in the absence or presence of an S9 fraction from rat liver used to mimic the metabolic function of mammalian systems in vivo. This may eventually disqualify this in vitro method from being a completely non-animal procedure [35]. However, the use of in vitro methods (NAMs) that include metabolic activation still significantly reduces the number of laboratory animals needed and provides testing with higher human relevance.

With regard to the European Commission’s call for data submission, we have selected a battery of in vitro toxicological methods, namely the Ames Test (MPF™ Test, Xenometrix, OECD TG 471) with *S. typhimurium* strains TA98, TA100, TA1535 and TA1537; the Comet assay in vitro performed on the HaCat cell line (non-tumor human keratinocytes); the Mammalian Chromosome Aberration Test on human peripheral lymphocytes (OECD TG 473); and XenoScreen YES/YAS assay (Xenometrix, Allschwil, Switzerland). We used these assays (NAMs) in combination to screen TCS and TCC for genotoxic and mutagenic properties, and their potential for endocrine disruption, applying the most feasible non-animal approach.

## 2. Materials and Methods

### 2.1. Chemicals

**Figure 1:**
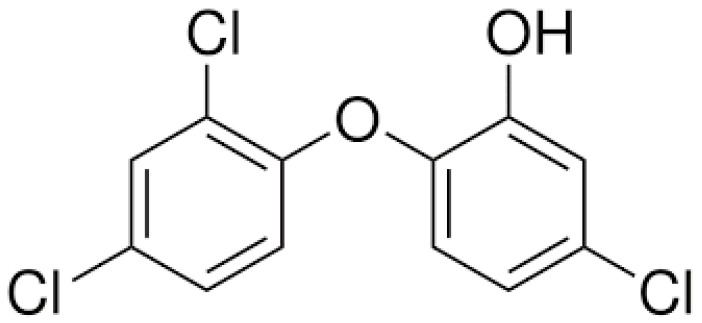
Chemical structure of Triclosan.

**Figure 2:**
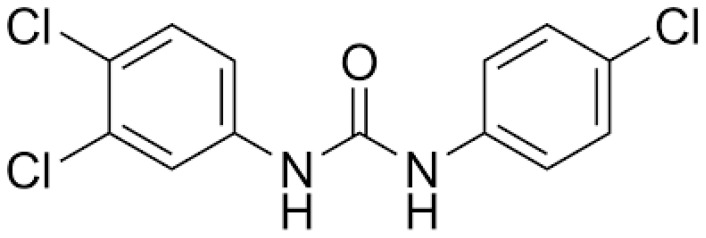
Chemical structure of Triclocarban.

Triclocarban, (CAS No.: 101-20-2, EC No.: 202-924-1, IUPAC name: N-(4-Chlorophenyl)-N′-(3,4-dichlorophenyl)urea), (TCC), and Triclosan, (CAS No.: 3380-34-5, EC No.: 222-182-2, IUPAC Name: 5-chloro-2-(2,4-dichlorophenoxy)phenol), (TCS), were supplied by Sigma-Aldrich Co., St. Louis, MI, USA, and used as test substances. Their chemical structures are depicted in Figure 1 and Figure 2.

### 2.2. Metabolic Activation

An exogenous source of the metabolic activation system (MAS), i.e., a cofactor-supplemented post-mitochondrial fraction prepared from rodent livers (Wistar rats, males, 7–8 weeks old) treated with an enzyme-inducing agent (Aroclor 1254 or Phenobarbital/β-Naphtoflavone), was used to model mammalian metabolic activation. This production was conducted at the National Institute of Public Health, Prague, Czech Republic, under project No. MZDR 37519/2019-4/OVZ, and approved by the Czech Ministry of Health.

### 2.3. Methods

#### 2.3.1. Bacterial Reverse Mutation Test (Ames MPF™ Test)

Ames MPF™ Test (Xenometrix AG, Allschwil, Switzerland) is a commercially available bacterial-modified bacterial plate method, based on the procedure described in OECD TG 471 [36], used for identifying compounds with genotoxic activity. The assay was conducted following the provided standard operating procedure (SOP) and utilized standardized materials and chemicals [37]. Four *Salmonella typhimurium* strains (TA98, TA100, TA1535, TA1537) were employed. All positive control chemicals (2-nitrofluorene (2-NF), 4-nitroquinoline N-oxide (4-NQO), N4-aminocytidine (N4-ACT) and 9-aminoacridine (9-AAc)) for tests with the *S. typhimurium* strains without S9 (S9−) and 2-aminoanthracene (2-AA) for tests in the presence of S9 (S9+) were supplied with the kit. The rat liver S9 fraction, including S9 100/1537 Booster solution, was also provided and was specific for each *S. typhimurium* strain with or without metabolic activation (S9+/S9−), as specified in the supplied SOP. Growth medium was used as the negative control. Briefly, 10 mL growth medium was mixed with 25 μL of the frozen stock culture of the tested strains (TA98 and TA100) in the presence of 10 μL ampicillin (Xenometrix). The strains were incubated at 37 °C in a shaking incubator (NB-205, N-BIOTEK) at 250 rpm for 14–16 h, overnight. The culture density was verified by measuring OD at 600 nm (BioTek Eon High Performance Microplate Spectrophotometer, Agilent Technologies, Santa Clara, CA, USA). The required OD values for the cultures to be suitable for testing should be at least 2.0, and for the negative control (growth medium without culture), should be <0.05. For testing, 10 μL aliquots of test items were added to plates S9−/S9+ with a final concentration of S9 fraction of 4.5%. Three pre-screened non-cytotoxic concentrations were evaluated in triplicate. Then, 240 μL/well of the suspension was transferred to 24-well plates, sealed with breathable tapes and incubated at 37 °C for 90 min with shaking at 250 rpm. After 90 min of incubation, a reversion indicator medium (Xenometrix) was dispensed into each well of the 24-well plates. The indicator medium was gently mixed, and 50 μL/well was transferred to a 384-well microtiter plate. Each column of the 24-well plate was transferred into one half of a 384-well microtiter plate, effectively dividing each sample among 48 wells. Three plates were used per strain with/without S9 (S9+/S9−). The 384-well microtiter plates were placed in plastic bags to reduce evaporation and were incubated at 37 °C for approximately 48 h. The average number of triplicate wells containing revertants per culture/dose was calculated. The combined criteria ‘Fold increase (FI) over baseline ≥ 2’ and ‘Binomial B-value ≥ 0.99’ were used to score individual doses as positive (software provided by the kit manufacturer Xenometrix: YES/YAS Calculation Workbook in MS Excel, created by M. Kamber, Xenometrix AG, 2012).

#### 2.3.2. In Vitro Mammalian Chromosome Aberration Test

OECD TG 473 [38] was followed as the main methodological guideline. Peripheral blood lymphocytes were used as the test system. A metabolic activation system (S9 liver fraction), i.e., a cofactor-supplemented post-mitochondrial fraction prepared from rodent livers (Wistar rats, males, 7–8 weeks old) treated with an enzyme-inducing agent (Aroclor 1254 or Phenobarbital/β-Naphtoflavone), was employed to model mammalian metabolic activation. After 48 h of cultivation, the test system was exposed to the test chemicals at previously selected non-cytotoxic concentrations: with or without metabolic activation (+/− S9), positive controls (Thio-TEPA 10^−6^ M without S9, Sigma Aldrich, Germany; Cyclophosphamide 10^−4^ M with S9, Bristol Myers Squibb, USA) and negative control (culture medium). The exposure period was 4 h with or without S9 (+/− S9) and 26 h without S9 (−S9). Following the exposure period, the test system was exposed to Colchicine for 2 h (Sigma Aldrich, Darmstadt, Germany) to arrest the cell cycle in metaphase. The cell cultures were harvested and subjected to hypotonic treatment and fixation on glass slides. Samples on glass slides were stained with 5% Giemsa (Penta, Slovakia, Czech Republic). At least 200 metaphase cells with well-spaced 46 +/−2 centromeres were subjected to microscopic analysis and evaluated for the frequency of chromosome aberrations (i.e., the percentage of metaphase cells with chromosome aberrations). The criterion for evaluating a result as positive was at least a 2-fold increase in the number of cells with chromosome aberrations compared to the number observed for the negative control. The test was performed at least in duplicate.

#### 2.3.3. In Vitro Comet Assay

OECD TG 489 [39] was followed as the main methodological guideline with modifications as follows: a human relevant cell line derived from human keratinocytes (HaCaT cell line, ATCC, USA) was used as the test system as described previously [40]. Test chemicals dissolved in culture medium (DMEM, Sigma Aldrich, Saint Louis, MO, USA) were applied to the 80% confluent cell culture monolayer at selected non-cytotoxic concentrations (24 h exposure period, 37 °C). Additionally, 1% H_2_O_2_ in PBS (15 min exposure period, 4 °C) was used as the positive control, and culture medium (DMEM, Sigma Aldrich, USA) was used as the negative control. After treatment, the morphology of the cells was microscopically inspected, and doses of test compounds with observed signs of cytotoxicity were excluded from further steps. TrypLE (Gibco, ThermoFisher Scientific, Waltham, MA, USA) was applied to the cell monolayer (for +/− 10 min) to gently harvest the cells. The cell suspension was centrifuged (6 min, 1000× *g*) and cell suspension samples were prepared by mixing the cell pellets with 85 µL of 1% low melting point (LMP) agarose (ThermoFisher, USA, 37 °C), dropping 85 µL of the LMP agarose cell suspension onto glass slides pre-coated with agarose gel (1% high melting point (HMP) agarose, ThermoFisher, USA) and covering with coverslips. Samples on glass slides with coverslips were solidified at 4 °C, and after removing the coverslips, the samples were lysed in a lysis buffer (1% Triton X-100, Serva, Heidelberg, Germany, 1 h, 4 °C) in glass Coplin Jars. After lysis, the agarose samples were subjected to pre-incubation in alkaline electrophoretic solution (4 °C, 40 min) in the electrophoretic tank (Sub-Cell Model 192 Cell, 2.000 mL). After pre-incubation, alkaline electrophoresis was performed (380 mA, 0.8 V cm^−1^, 20 min). After electrophoresis, the agarose samples were rinsed twice in a neutralization buffer for 10 min (0.4 M Tris, pH 7.5, 4 °C), stained (SYBR Green I, Invitrogen, Waltham, MA, USA) and subjected to microscopic evaluation (fluorescence microscope with a CCD camera, Olympus, Japan). The test was performed in triplicate, and the mean +/− standard deviation was calculated. At least 100 cells from each stained agarose sample were evaluated using automated software (CometScore 1.5, Sumerduck, VA, USA). The mean values of the amount of DNA in the head, the Olive momentum, and the amount of DNA in the tail of the comets were assessed (ANOVA, Dunnett *t*-tests, IBM SPSS Statistics for Windows, version 23.0 Armonk, NY, USA: IBM Corp.). The mean values of the DNA in the tail of the comets were used for interpretation. The criterion of a *p*-value < 0.05 was used as the threshold for statistically significant results. Provided that all acceptability criteria are fulfilled, a test chemical is considered to be clearly positive if: (a) at least one of the test doses exhibits a statistically significant increase compared with the concurrent negative control, (b) the increase is dose-related when evaluated with an appropriate trend test, (c) any of the results are outside the distribution of the historical negative control data for a given species, vehicle, route, tissue and number of administrations. When all of these criteria are met, the test chemical is then considered able to induce DNA strand breakage in the tissues studied in this test system. If only one or two of these criteria are satisfied (i.e., in the case the response is neither clearly negative nor clearly positive, and not all the criteria listed above are met), and in order to assist in establishing the biological relevance of a result, the data should be evaluated by expert judgement and/or further investigations should be conducted, if scientifically justified [39].

#### 2.3.4. XenoScreen^®^ YES/YAS Assay

Yeast Estrogen Screen/Yeast Androgen Screen (Xenometrix AG, Allschwil, Switzerland) is a yeast-based reporter gene assay designed for screening chemical compounds for estrogenic and androgenic agonistic/antagonistic activities, indicating endocrine disruption potential. The assay was performed in duplicate according to the manufacturer’s standard operating procedure [41], using supplied standardized chemicals. The positive controls used were: 17β-estradiol (10^−8^ M) in the agonistic (estrogenic) assay; 4-hydroxytamoxifen (10^−6^ M) in the antagonistic (anti-estrogenic) assay; 5α-dihydrotestosterone (10^−6^ M) in the agonistic (androgenic) assay; and flutamide (10^−8^ M) in the antagonistic (antiandrogenic) assay. Pre-cultured cell suspensions of two recombinant *Saccharomyces cerevisiae* strains expressing human estrogen (hERα) and androgen (hAR) receptors were prepared according to the manufacturer’s instructions, and were exposed to the test samples for 48 h on an orbital shaker (at 31 °C). The optical density (OD) of the red product resulting from the conversion of the yellow substrate after secretion of β-galactosidase was measured at 570 nm on BioTek Eon High Performance Microplate Spectrophotometer (BioTek, Agilent Technologies, Santa Clara, CA, USA). The OD570 of the end product, compared with the controls, directly correlates with the endocrine activity of the test samples. An increase of ≥10% in the absorbance value of the sample compared to that of the vehicle control (i.e., non-treated growth culture medium) was considered a potentially positive response in the agonistic assays (this was a precautionary and conservative evaluation). A decrease of ≥20% in the absorbance value compared to that of the negative control was considered a potentially positive response in the antagonistic assays, assuming a general biological variability of 20%.

## 3. Results

### 3.1. Bacterial Reverse Mutation Test (Ames MPF™ Test)

The Ames MPF™ Test was performed on four *S. typhimurium* strains, TA98, TA100, TA1535, TA1537, with and without metabolic activation (S9+/S9−) at three concentrations pre-screened for cytotoxicity and presumed to be non-toxic for the test system. The viable growing culture was visually inspected for turbidity/bacterial colonies in the wells, as recommended in the supplied SOP. However, TCS showed high cytotoxicity in all *S. typhimurium* strains without metabolic activation (S9−). Weak spontaneous mutations under 1% (SM < 1%) in strains TA98 and TA1537 with metabolic activation (S9+) were observed for TCS. Neither TCS nor TCC exhibited a clear concentration-dependent response in the mean number of revertant wells, nor did they show multiple positive responses with a fold increase (FI, fold inductions in revertant numbers over the baseline) ≥ 2 compared to the value of the baseline. At a concentration of 0.5 µg/mL, TCS tested with the *S. typhimurium* strain TA 1537 S9+ (*) showed an outlier value of FI ≥ 2, and this result has been concluded as unclear after being subjected to careful inspection and further evaluation. Fold inductions in revertant numbers over the baseline (FI) are generally not considered positive if they are less than 2.0. Below this FI value, the data are considered unreliable with respect to determining mutagenicity. A compound that shows a clear dose response and/or yields multiple FIs greater than 2.0 is classified as a mutagen. The result was not confirmed by further evaluation, as another observed FI value was ≤2 (0.5). The positive and negative control values and the values of the baseline were within the acceptable limits for an experiment to be considered valid. The mean value of the positive control for the *S. typhimurium* strain TA98 without metabolic activation (S9−) should be 40 at minimum. Thus, the value of 39.67 (**) observed in the TA98 strain was carefully inspected. As two out of three individual replicate values were ≥40, one replicate was considered as a borderline outlier (37), and the response of the positive control was accepted as being valid after an expert judgement and repeated testing within acceptable limits. None of the tested compounds were evaluated as being mutagenic in the Ames MPF™ Test (Table 1).

### 3.2. In Vitro Mammalian Chromosome Aberration Test (CA)

In the in vitro mammalian chromosome aberration test, both chemicals, TCS and TCC, were clearly positive at the highest tested pre-screened non-cytotoxic concentration (10 µg/mL) after treatment for 4 h (see Table 2). Prolonged exposure (26 h, without S9) at higher concentrations (5, 10 µg/mL) resulted in higher cytotoxicity, therefore, the data could not be evaluated as positive (NE = not evaluated).

### 3.3. In Vitro Comet Assay

In the in vitro Comet assay, the percentage of DNA in the tail (mean % of DNA in the tail ± SD) significantly increased at the highest tested non-cytotoxic concentrations of both tested preservatives, TCS and TCC, and a clear concentration-dependent positive response has been observed (see Table 3). The results (from three independent measurements) were considered statistically significant when the *p*-value was <0.05 (*).

### 3.4. XenoScreen^®^ YES/YAS Assay 

**Figure 3:**
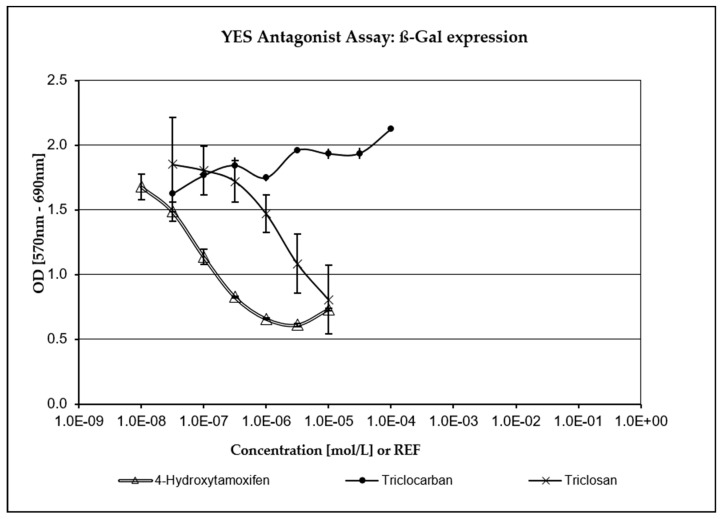
Anti-estrogenic activity. TCS exhibited a strong concentration-dependent response compared to the positive control (4-Hydroxytamoxifen).

**Figure 4:**
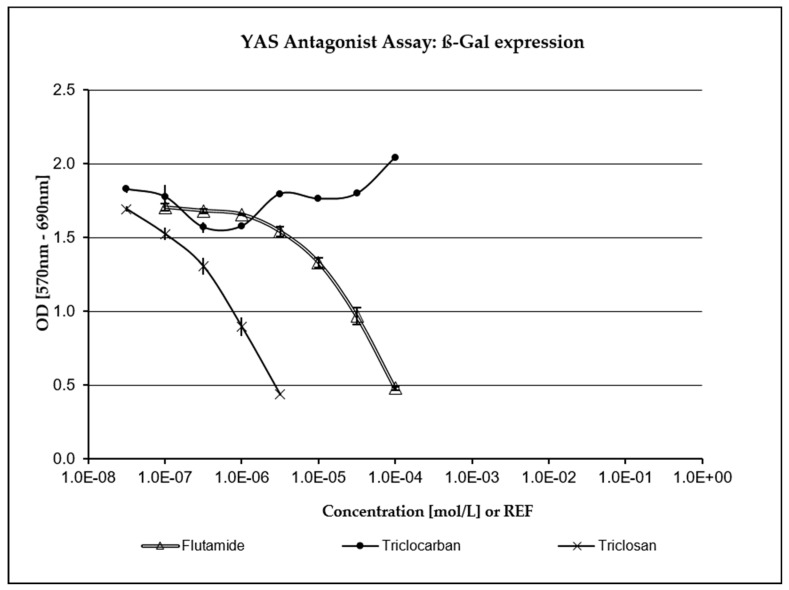
Anti-androgenic activity. TCS exhibited a strong concentration-dependent response compared to the positive control (Flutamide).

TCC has not been assessed as being positive for any endocrine activity in any of the XenoScreen^®^ assays included in the study and was not highly cytotoxic. As depicted in Figure 3 and Figure 4, TCS exhibited a strong concentration-dependent positive response in both the anti-estrogenic assay and the anti-androgenic assay (where it demonstrated even greater potency than the positive control, Flutamide). Fungicidal activity against *S. cerevisiae* was carefully monitored during the study through repeated turbidity measurements of the culture at OD690. TCS has demonstrated significant cytotoxic (fungicidal) effects compared to the positive control and the negative (vehicle) control, as well as TCC (which showed only mild cytotoxicity in *S. cerevisiae* strain hERα within acceptable limits). Only five non-cytotoxic concentrations of TCS were deemed to be acceptable for evaluating endocrine-disrupting activity in the transfected *S. cerevisiae* strain hAR with the incorporated human androgen receptor (hAR) (i.e., 3.16 × 10^−8^–3.16 × 10^−6^ M). Additionally, only six non-cytotoxic concentrations of TCS were evaluated in the transfected *S. cerevisiae* strain with the incorporated human estrogen receptor α (hERα) (3.16 × 10^−8^–1.06 × 10^−5^ M).

## 4. Discussion

In publicly available registration dossiers [42,43], in the opinions of the Scientific Committee for Consumer Products [44] and in recent scientific literature [45], both TCS and TCC have consistently produced negative results in the Ames test performed according to OECD TG 471 [36]. These findings align with the results of our study, utilizing the modified microplate Ames MPF™ test. In our study, a higher cytotoxicity was observed in all *S. typhimurium* strains tested without metabolic activation (S9−) for TCS compared to TCC. It is likely that the antimicrobial activity of TCS was inhibited by enzymes contained in the liver homogenate (S9 fraction), or that TCS was converted into metabolites with lower cytotoxicity. This underscores the justification of including S9 in toxicological methods in vitro to ensure higher human relevance regarding the metabolic conversion of xenobiotics in the liver and the toxicokinetics of the organism, considering its absorption, distribution, metabolism and excretion in vivo. We suggest carefully considering cytotoxicity data in any in vitro assay, as false positive/negative results may be obtained, especially when testing highly cytotoxic antimicrobials. We argue that testing antimicrobials using the Ames test as a stand-alone in vitro assay cannot provide reliable information on the mutagenic potency of the test substance in mammals (eukaryotes). Therefore, the obtained results should be confirmed in other biological testing more relevant to humans and less sensitive to antimicrobial activity. However, neither TCS nor TCC showed any potential for mutagenicity in the modified microplate Ames MPF^TM^ test. This aligns with previous results from recent studies on the classical plate-format Ames test, where treatment with 10 μg/plate TCS also produced inhibitory effects, while 1 and 0.1 μg/plate TCS did not markedly affect the number of colonies or frequency of mutants in *S. typhimurium* strains [46]. Historical data from the classical Ames test, which used *S. typhimurium* and the yeast *S. cerevisiae*, are included in the registration dossier for TCS in the ECHA database, and they consistently generated negative results [42].

For TCC, the data on genotoxicity are scarce, however, recent studies have consistently produced negative results, and TCC was evaluated as being non-genotoxic, as supported by data available in the registration dossier of the ECHA database [2,43,45].

In the mammalian chromosome aberration test, both TCS and TCC tested positive at the highest non-cytotoxic concentration in our study (Table 2). Prolonged exposure (26 h, without S9) resulted in higher cytotoxicity, therefore, the data could not be accepted for evaluation. Although the majority of the scarce results available in the ECHA registration dossiers classified both substances as non-genotoxic, a concentration-related increase in chromosomal aberrations in Chinese Hamster V79 cells exposed to the highest concentration of TCS (3.0 μg/mL) at the 18 h and 28 h fixation intervals without S9 was observed [47]. This observation aligns with our positive results generated on a mammalian-relevant test system (human peripheral lymphocytes). The relatively limited number of studies available in the ECHA database or in the SCCS reports provided rather sparse data on chromosome aberrations induced by TCS, which tested negative in assays with CHO cells or in the micronucleus test using mice (bone marrow) [44,48]. A significant (*p* ≤ 0.05) concentration-dependent increase in aberrant cells was observed after *Labeo rohita* hatchlings were exposed to TCS for 96 h (supported by a concentration-dependent increase in necrotic, apoptotic and micronucleated cells) [49]. Nonactivated TCS was found to induce a dose-related increase in the yield of cells with abnormal chromosome morphology in the in vitro mammalian chromosomal aberration test with dose levels ranging from 1 to 3 μg/mL (18 h harvest) and at 3 μg/mL (28 h harvest). The most frequently observed type of chromosome damage was exchange figures. However, no signs of structural chromosomal aberrations were observed in the in vivo bone marrow chromosomal aberration test [50].

In the case of TCC, data on induced chromosomal aberrations are even more scarce. As concluded in the “Screening Assessment of Urea, N-(4-chlorophenyl)-N’-(3,4-dichlorophenyl) (Triclocarban)” published by the Government of Canada in March 2023 [51], TCC tested negative in an in vitro chromosome aberration test in Chinese hamster ovary cells, both with and without metabolic activation, at concentrations up to 2000 µg/mL [52]. In Tox21 assays, TCC was identified as genotoxic in cell lines deficient in DNA repair pathways [53]. The limited data available have been evaluated by the Scientific Committee on Consumer Safety, concluding that TCC was not found to be clastogenic in the chromosomal aberration test with and without metabolic activation [2].

In the Comet assay performed on the HaCaT cell line, the percentage of DNA in the tail was statistically significantly increased at the two highest tested concentrations upon exposure to TCS (5, 10 µg/mL) and at all three tested concentrations upon exposure to TCC (2.5, 5, 10 µg/mL). Such results are supported, for example, by a study using aquatic larvae of the insect *Chironomus riparius*, where TCS was found to have genotoxic activity as it significantly increased all the comet parameters (% DNA in tail, tail length, tail moment, Olive tail moment) at all tested concentrations [54], or in a study of the binary combination of TCS and carbendazim tested on *Daphnia magna* [55]. Both TCS and TCC caused concentration-dependent DNA damage to the protozoan *Tetrahymena thermophila* in the alkaline Comet assay, with TCC inducing more severe DNA damage than TCS [56]. However, another study performed on HaCaT and L02 cells treated with TCC provided negative results [46].

As stated in the OECD TG 489, positive findings in the Comet assay may not be solely associated with genotoxicity. Target tissue toxicity may also result in increased DNA migration. Low or moderate cytotoxicity is often observed, making it challenging to distinguish DNA migration induced by genotoxicity versus DNA migration induced by cytotoxicity in the Comet assay alone. However, when increases in DNA migration are observed, performing an examination of one or more indicators of cytotoxicity is recommended, as this can aid in interpreting the findings. Increases in DNA migration in the presence of clear evidence of cytotoxicity should be interpreted with caution [39,57,58].

The growing evidence for the genotoxic effects of TCS and TCC on aquatic organisms and fish [59,60,61,62] supports warnings about TCS and TCC and raises efforts to clarify the mechanisms by which the effects of these polychlorinated preservatives occur in more detail, including oxidative damage in specific cells and tissues [63,64,65,66,67], potential synergistic effects with other substances present in final cosmetic products or the environment [55,68,69,70] and evaluations of the safety of their active metabolites, products of biodegradation or photolysis, considering their widespread use in consumer products and their presence in the environment [1,71]. Numerous inconclusive data and variability in test systems support the need to employ test systems highly relevant to humans, i.e., human-derived cell lines or 3D reconstructed human tissue models should be preferably used for safety assessment in vitro.

The potential for endocrine disruption elicited by TCS and TCC was examined in this study using the commercially available in vitro test, XenoScreen YES/YAS (Xenometrix, Switzerland). Severe cytotoxicity and the highly positive response of TCS were observed at five low, non-cytotoxic concentrations in both antagonistic assays conducted (anti-estrogenic assay, anti-androgenic assay). This was compared to the relevant positive controls (4-Hydroxytamoxifen in the anti-estrogenic assay, Flutamide in the anti-androgenic assay) and the vehicle control (culture medium providing a negative response). Cytotoxicity data were considered, and only non-cytotoxic concentrations were selected for the evaluation of endocrine activity. TCC did not show any conclusive positivity or cytotoxicity in the XenoScreen YES/YAS assay. The anti-estrogenic and anti-androgenic behavior of TCS was also confirmed in another recent study utilizing XenoScreen YES/YAS assay [72]. It was reported that TCC exhibited agonist activity on the androgen receptor and estrogen receptor alpha, and antagonist activity on glucocorticoid and the thyroid receptor. TCS showed antagonist effects on the androgen receptor, estrogen receptor alpha, glucocorticoid and the thyroid receptor [73].

Recent public concerns regarding the potential toxicological effects and environmental accumulation of TCS and TCC have led to efforts to find efficient replacements for these antimicrobials [1], such as pentafluorosulfanyl-containing Triclocarban analogs [74] or benzalkonium chloride, benzethonium chloride and chloroxylenol. However, these substances have not been tested as extensively as TCS and TCC and may, therefore, pose a greater risk to humans or the environment [75]. Further investigation is required to understand their mechanisms of action and ecological significance before their introduction into widespread use in consumer products can be conclusively justified.

## 5. Conclusions

The potential for mutagenicity, genotoxicity and endocrine disruption in Triclosan and Triclocarban was assessed using NAMs, representing the most suitable non-animal in vitro approach for testing cosmetic ingredients in the EU. The results suggest that both chemicals may exhibit a potential for genotoxicity, depending on the specific in vitro method used, highlighting the advantageous use of NAMs in combination. Triclosan demonstrated some potential for endocrine disruption, specifically anti-estrogenic and anti-androgenic effects. Our study aimed to enhance the toxicological profiles of both preservatives, as there was a lack of in vitro data generated using recently implemented NAMs of higher human relevance, validated or sufficiently standardized for testing cosmetic ingredients. We believe that, in response to the recent European Commission call for data, the provided results could contribute to discussions and the amendment of regulatory measures aimed at enhancing the safety of cosmetic ingredients within consumer products. Our study seeks to illustrate that NAMs based on human-relevant cell lines or transfected microorganisms can yield valuable results when employed in combination. The broader implementation of human-relevant, validated and standardized NAMs should be encouraged, considering their greater acceptance in human risk assessment for regulatory purposes. 

## Figures and Tables

**Table 1:** Results of the Ames MPF™ Test (Xenometrix, Switzerland). *S. typhimurium* strains TA98, TA100, TA1535, TA1537 were tested with and without metabolic activation (S9+/S9−). Mean numbers of revertant wells from triplicates are presented for the tested concentrations of TCS (0.5–1.0–2 µg/mL) and TCC (2.5–5.0–10 µg/mL), negative control (NC, culture growth media) and positive control chemicals (PC) specific for each *S. typhimurium* strain with or without S9. A fold increase (FI, inductions in the number of revertant wells) greater than 2.0 at multiple concentrations is required for a result to be evaluated as being positive for mutagenicity. The mean numbers of revertant colonies of the positive control chemicals (PC) are proposed in the supplied SOP to have a threshold of >3-fold as the baseline for TA98, TA1535 and TA1537, and >2-fold as the baseline for TA100. The presented data were generated in two separate runs, in triplicate.

Triclosan								
*S. typhimurium* strain	TA 98		TA 100		TA 1535		TA 1537	
S9 −/+	S9−	S9+	S9−	S9+	S9−	S9+	S9−	S9+
0.5 µg/mL	0.00	4.33	0.00	3.33	0.00	0.33	0.00	2.00
FI	0.00	0.84	0.00	0.48	0.00	0.33	0.00	2.00 *
1.0 µg/mL	0.00	6.00	0.00	2.00	0.00	0.00	0.00	1.33
FI	0.00	1.16	0.00	0.29	0.00	0.00	0.00	1.33
2.0 µg/mL	0.00	6.67	0.00	0.00	0.00	0.67	0.00	0.33
FI	0.00	1.29	0.00	0.00	0.00	0.67	0.00	0.33
NC	2.67	2.67	4.00	4.00	0.33	0.33	1.33	0.33
Baseline	5.18	5.18	7.00	7.00	1.00	1.00	2.86	1.00
PC	39.67 **	48.00	48.00	48.00	48.00	48.00	48.00	44.00
FI	7.65	9.26	6.86	6.86	48.00	48.00	16.78	44.00
**Triclocarban**								
***S. typhimurium* strain**	**TA 98**		**TA 100**		**TA 1535**		**TA 1537**	
S9 −/+	S9−	S9+	S9−	S9+	S9−	S9+	S9−	S9+
2.5 µg/mL	0.00	4.00	1.67	5.33	0.00	0.67	2.67	2.33
FI	0.00	0.64	0.19	0.91	0.00	0.14	0.89	1.17
5.0 µg/mL	2.33	4.33	3.33	4.67	0.67	1.00	2.33	1.33
FI	0.39	0.69	0.37	0.80	0.33	0.21	0.78	0.67
10.0 µg/mL	3.33	4.33	2	5.67	0.00	0.67	1.00	1.67
FI	0.56	0.69	0.22	0.97	0.00	0.14	0.33	0.83
NC	3.67	5.67	9.00	3.33	1.00	3.00	3.00	2.00
Baseline	5.98	6.24	9.00	5.85	2.00	4.73	3.00	2.00
PC	41.00	40.67	47.33	42.67	44.33	39.67	45.67	37.67
FI	6.86	6.51	5.26	7.29	22.17	8.38	15.22	18.83

**Table 2:** In vitro mammalian chromosome aberration test (% aberrant cells, 4 h +/− S9, 26 h –S9, NE—non-evaluated).

Samples/ Controls	Concentration µg/mL	% Abberant Cells			Evaluation
		4 h + S9	4 h − S9	26 h − S9	
**Triclosan**	2.5	5	6	4	negative
	5	4	5	NE	negative
	10	21	10	NE	positive
**Triclocarban**	2.5	4	3	3	negative
	5	7	7	NE	borderline
	10	12	8	NE	positive
Negative control 1 (culture medium)		4	3	4	negative
Positive control 1 (Thio-TEPA, 10^−6^ M)			9	10	positive
Negative control 2 (+S9)		3			negative
Positive control 2 (cyclophosphamide 10^−4^ M)		10			positive

**Table 3:** In vitro Comet assay, HaCaT cell line. % DNA in tail (mean ± SD values). (*) statistically significant result with *p* < 0.05.

Samples/ Controls	Concentration µg/mL	% DNA in Tail Mean ± SD	*p*-Value
**Triclosan**	2.5	3.75 ± 0.34	**0.060**
	5	13.87 ± 1.51 *	**<0.0001**
	10	15.99 ± 1.64 *	**<0.0001**
**Triclocarban**	2.5	6.08 ± 0.20 *	**0.006**
	5	7.86 ± 0.98 *	**0.001**
	10	11.92 ± 2.28 *	**0.0005**
**Negative control** **(culture medium)**		0.63 ± 0.07	**-**
**Positive control** **(1% H_2_O_2_)**		91.48 ± 0.75	**-**

## Data Availability

The data presented in this study are available on request from the corresponding author. The data are not publicly available due to the copyright policy of the National Institute of Public Health and the grant project regulations.

## References

[1] Iacopetta D., Catalano A., Ceramella J., Saturnino C., Salvagno L., Ielo I., Drommi D., Scali E., Plutino M.R., Rosace G. (2021). The Different Facets of Triclocarban: A Review. Molecules.

[2] SCCS (Scientific Committee on Consumer Safety) (2022). Request for a Scientific Advice on the Safety of Triclocarban (CAS No. 101-20-2, EC No. 202-924-1) and Triclosan (CAS No. 3380-34-5, EC No. 222-182-2) as Substances with Potential Endocrine Disrupting Properties Used in Cosmetic Products, Preliminary Version of 15–16 March 2022, final version of 24–25 October 2022, SCCS/1643/22. https://health.ec.europa.eu/system/files/2023-08/sccs_o_265.pdf.

[3] Shrestha P., Zhang Y., Chen W.J., Wong T.Y. (2020). Triclosan: Antimicrobial mechanisms, antibiotics interactions, clinical applications, and human health. J. Environ. Sci. Health C Toxicol. Carcinog..

[4] Zhang H., Li J., An Y., Wang D., Zhao J., Zhan M., Xu W., Lu L., Gao Y. (2022). Concentrations of bisphenols, benzophenone-type ultraviolet filters, triclosan, and triclocarban in the paired urine and blood samples from young adults: Partitioning between urine and blood. Chemosphere.

[5] Pycke G.F.B., Geer A.L., Dalloul M., Abulafia O., Jenck M.A., Halden U.R. (2014). Human fetal exposure to triclosan and triclocarban in an urban population from Brooklyn, New York. Environ. Sci. Technol..

[6] Wei L., Qiao P., Shi Y., Ruan Y., Yin J., Wu Q., Shao B. (2017). Triclosan/triclocarban levels in maternal and umbilical blood samples and their association with fetal malformation. Clin. Chim. Acta.

[7] Toms L.M.L., Allmyr M., Mueller J.F., Adolfsson-Erici M., McLachlan M., Murby J., Harden F.A. (2011). Triclosan in individual human milk samples from Australia. Chemosphere.

[8] Kim J.H., Kim D., Moon S.M., Yang E.J. (2020). Associations of lifestyle factors with phthalate metabolites, bisphenol A, parabens, and triclosan concentrations in breast milk of Korean mothers. Chemosphere.

[9] Asimakopoulos A.G., Thomaidis N.S., Kannan K. (2014). Widespread occurrence of bisphenol A diglycidyl ethers, p-hydroxybenzoic acid esters (parabens), benzophenone type-UV filters, triclosan, and triclocarban in human urine from Athens, Greece. Sci. Total Environ..

[10] Xue J., Wu Q., Sakthivel S., Pavithran V.P., Vasukutty R.J., Kannan K. (2015). Urinary levels of endocrine-disrupting chemicals, including bisphenols, bisphenol A diglycidyl ethers, benzophenones, parabens, and triclosan in obese and non-obese Indian children. Environ. Res..

[11] Iyer P.A., Xue J., Honda M., Robinson M., Kumosami A.T., Abulnaja K., Kannan K. (2018). Urinary levels of triclosan and triclocarban in several Asian countries, Greece and the USA: Association with oxidative stress. Environ. Res..

[12] Li W., Zhang W., Chang M., Ren J., Xie W., Chen H., Zhang Z., Zhuang X., Shen G., Li H. (2018). Metabonomics reveals that triclocarban affects liver metabolism by affecting glucose metabolism, β-oxidation of fatty acids, and the TCA cycle in male mice. Toxicol. Lett..

[13] Tian X., Huang K., Liu Y., Jiang K., Liu R., Cui J., Wang F., Yu Y., Zhang H., Lin M. (2023). Distribution of phthalate metabolites, benzophenone-type ultraviolet filters, parabens, triclosan and triclocarban in paired human hair, nail and urine samples. Environ. Pollut..

[14] Yin J., Wei L., Shi Y., Zhang J., Wu Q., Shao B. (2016). Chinese population exposure to triclosan and triclocarban as measured via human urine and nails. Environ. Geochem. Health.

[15] Armstrong D.L., Lozano N., Rice C.P., Ramirez M., Torrents A. (2018). Degradation of triclosan and triclocarban and formation of transformation products in activated sludge using benchtop bioreactors. Environ. Res..

[16] Chen J., Meng X.Z., Bergman A., Halden R.U. (2019). Nationwide reconnaissance of five parabens, triclosan, triclocarban and its transformation products in sewage sludge from China. J. Hazard. Mater..

[17] Meador J.P., Yeh A., Young G., Gallagher E.P. (2016). Contaminants of emerging concern in a large temperate estuary. Environ. Pollut..

[18] Gomes M.F., de Paula V.D.C.S., Martins L.R.R., Garcia J.R.E., Yamamoto F.Y., de Freitas A.M. (2021). Sublethal effects of triclosan and triclocarban at environmental concentrations in silver catfish (*Rhamdia quelen*) embryos. Chemosphere.

[19] Lozano N., Rice C.P., Ramirez M., Torrents A. (2018). Fate of triclocarban in agricultural soils after biosolid applications. Environ. Sci. Pollut. Res..

[20] Vimalkumar K., Seethappan S., Pugazhendhi A. (2019). Fate of Triclocarban (TCC) in aquatic and terrestrial systems and human exposure. Chemosphere.

[21] Yang H., Sanidad K.Z., Wang W., Xie M., Gu M., Cao X., Xiao H., Zhang G. (2020). Triclocarban exposure exaggerates colitis and colon tumorigenesis: Roles of gut microbiota involved. Gut Microbes.

[22] Wu Y., Beland F.A., Fang J.L. (2016). Effect of triclosan, triclocarban, 2,2′,4,4′-tetrabromodiphenyl ether, and bisphenol A on the iodide uptake, thyroid peroxidase activity, and expression of genes involved in thyroid hormone synthesis. Toxicol. Vitr..

[23] Rochester J.R., Bolden A.L., Pelch K.E., Kwiatkowski C.F. (2017). Potential developmental and reproductive impacts of triclocarban: A scoping review. J. Toxicol..

[24] Aker A.M., Ferguson K.K., Rosario Z.Y., Mukherjee B., Alshawabkeh A.N., Cordero J.F., Meeker J.D. (2019). The associations between prenatal exposure to triclocarban, phenols and parabens with gestational age and birth weight in northern Puerto Rico. Environ. Res..

[25] Cao L.Y., Xu Y.H., He S., Ren X.M., Yang Y., Luo S., Xie X.D., Luo L. (2020). Antimicrobial triclocarban exhibits higher agonistic activity on estrogen-related receptor γ than triclosan at human exposure levels: A novel estrogenic disruption mechanism. Environ. Sci. Technol. Lett..

[26] Costa N.O., Forcato S., Cavichioli A.M., Pereira M.R.F., Gerardin D.C.C. (2020). In utero and lactational exposure to triclocarban: Age-associated changes in reproductive parameters of male rat offspring. Toxicol. Appl. Pharmacol..

[27] Xie M., Zhang H., Wang W., Sherman H.L., Minter L.M., Cai Z., Zhang G. (2020). Triclocarban exposure exaggerates spontaneous colonic inflammation in Il-10^−/−^ mice. Toxicol. Sci..

[28] Sanidad K.Z., Wang G., Panigrahy A., Zhang G. (2022). Triclosan and triclocarban as potential risk factors of colitis and colon cancer: Roles of gut microbiota involved. Sci. Total Environ..

[29] Giuliano C.A., Rybak M.J. (2015). Efficacy of triclosan as an antimicrobial hand soap and its potential impact on antimicrobial resistance: A focused review. Pharmacotherapy.

[30] Hartmann E.M., Hickey R., Hsu T., Betancourt Roman C.M., Chen J., Schwager R., Kline J., Brown G.Z., Halden R.U., Huttenhower C. (2016). Antimicrobial chemicals are associated with elevated antibiotic resistance genes in the indoor dust microbiome. Environ. Sci. Technol..

[31] Zhang D., Lu S. (2023). A holistic review on triclosan and triclocarban exposure: Epidemiological outcomes, antibiotic resistance, and health risk assessment. Sci. Total Environ..

[32] Westfall C., Flores-Mireles A.L., Robinson J.I., Lynch A.J.L., Hultgren S., Henderso J.P., Levin P.A. (2019). The widely used antimicrobial Triclosan induces high levels of antibiotic tolerance i*n vitro* and reduces antibiotic efficacy up to 100-fold in vivo. Antimicrob. Agents Chemother..

[33] European Union (2009). Regulation EC No. 1223/2009 of the European Parliament and of the Council of 30 November 2009 on Cosmetic Products (Recast) (Text with EEA Relevance). Off. J. Eur. Union.

[34] FDA (U.S. Food and Drug Administration) (2016). Safety and Effectiveness of Consumer Antiseptics. Topical Antimicrobial Drug Products for Over-the-Counter Human Use. Final Rule. Fed. Reg..

[35] Cartus A., Schrenk D. (2016). Current methods in risk assessment of genotoxic chemicals. Food Chem. Toxicol..

[36] (2020). OECD Test No. 471: *Bacterial Reverse Mutation Test*, OECD Guidelines for the Testing of Chemicals, Section 4, OECD Publishing, Paris. https://www.oecd-ilibrary.org/environment/test-no-471-bacterial-reverse-mutation-test_9789264071247-en.

[37] Xenometrix (2019). Ames MPF^TM^ Penta 1 Microplate Format Mutagenicity Assay. Instructions for Use.

[38] (2016). OECD Test No. 473: *In Vitro Mammalian Chromosomal Aberration Test*, OECD Guidelines for the Testing of Chemicals, Section 4, OECD Publishing, Paris. https://www.oecd-ilibrary.org/environment/test-no-473-in-vitro-mammalian-chromosomal-aberration-test_9789264264649-en.

[39] (2016). OECD Test No. 489: *In Vivo Mammalian Alkaline Comet Assay*, OECD Guidelines for the Testing of Chemicals, Section 4, OECD Publishing, Paris. https://www.oecd-ilibrary.org/environment/test-no-489-in-vivo-mammalian-alkaline-comet-assay_9789264264885-en.

[40] Jiravova J., Tomankova K., Harvanova M., Malina L., Malohlava J., Luhova L., Panacek A., Manisova B., Kolarova H. (2016). The effect of silver nanoparticles and silver ions on mammalian and plant cells in vitro. Food Chem. Toxicol..

[41] Xenometrix AG (2017). XenoScreen YES/YAS Instructions for Use.

[42] European Chemical Agency ECHA: Triclosan Dossier. https://echa.europa.eu/cs/registration-dossier/-/registered-dossier/12675/7/7/1.

[43] European Chemical Agency ECHA: Triclocarban Dossier. https://echa.europa.eu/cs/registration-dossier/-/registered-dossier/12075/7/7/2.

[44] SCCP (Scientific Committee on Consumer Products) Opinion on Triclosan, 21 January 2009, SCCP/1192/08. https://ec.europa.eu/health/ph_risk/committees/04_sccp/docs/sccp_o_166.pdf.

[45] Sun D., Zhao T., Wang T., Wu M., Zhang Z. (2020). Genotoxicity assessment of triclocarban by comet and micronucleus assays and Ames test. Environ. Sci. Pollut. Res..

[46] Sun D., Zhao T., Li X., Zhang Z. (2019). Evaluation of DNA and chromosomal damage in two human HaCaT and L02 cells treated with varying triclosan concentrations. J. Toxicol. Environ. Health A.

[47] Heidemann A. (1990). Chromosome aberration assay in Chinese Hamster V79 cells in vitro with FAT 80′ 023/Q. Cytotest Cell Res..

[48] SCCS (Scientific Committee on Consumer Safety) Opinion on Triclosan, ADDENDUM to the SCCP Opinion on Triclosan (SCCP/1192/08) from January 2009, 22 March 2011, SCCS/1414/11. https://ec.europa.eu/health/scientific_committees/consumer_safety/docs/sccs_o_054.pdf.

[49] Sharma S., Dar O.I., Andotra M., Sharma S., Bhagat A., Thakur S., Kesavan A.K., Kaur A. (2022). Cellular, molecular and genomic alterations in the hatchlings of *Labeo rohita* after exposure to Triclosan. Front. Environ. Sci..

[50] US Environmental Protection Agency (2008). 5-Chloro-2-(2,4-dichlorophenoxy)phenol (Triclosan): Toxicology Chapter for the Reregistration Eligibility Decision (RED) Document.

[51] Government of Canada (2023). Screening Assessment Urea, N-(4-chlorophenyl)-N’-(3,4-dichlorophenyl)-(Triclocarban). Chemical Abstracts Service Registry Number 101-20-2. Environment and Climate Change Canada. Health Canada, March 2023, Cat. No.: En84-317/2022E-PDF. https://publications.gc.ca/collections/collection_2023/eccc/En84-317-2022-eng.pdf.

[52] Soap and Detergent Association (2002). In vitro Mammalian Chromosome Aberration Test. Report no. 2002-01-TCC. https://www.cleaninginstitute.org/sites/default/files/research-pdfs/Triclocarban_in_vitro_mammalian_chromosome_aberration_test.pdf.

[53] Kim S., Chen J., Cheng T., Gindulyte A., He J., He S., Li Q., Shoemaker B.A., Thiessen P.A., Yu B. (2019). PubChem 2019 update: Improved access to chemical data. Nucleic Acids Res..

[54] Martínez-Paz P., Morales M., Martínez-Guitarte J.L., Morcillo G. (2013). Genotoxic effects of environmental endocrine disruptors on the aquatic insect *Chironomus riparius* evaluated using the comet assay. Mutat. Res. Genet. Toxicol. Environ. Mutagen..

[55] Silva A.R., Cardoso D.N., Cruz A., Lourenço J., Mendo S., Soares A.M., Loureiro S. (2015). Ecotoxicity and genotoxicity of a binary combination of triclosan and carbendazim to *Daphnia magna*. Ecotoxicol. Environ. Saf..

[56] Gao L., Yuan T., Cheng P., Bai Q., Zhou C., Ao J., Wang W., Zhang H. (2015). Effects of triclosan and triclocarban on the growth inhibition, cell viability, genotoxicity and multixenobiotic resistance responses of *Tetrahymena thermophila*. Chemosphere.

[57] (2014). OECD Report of the JaCVAM Initiative International Pre-Validation and Validation Studies of the In Vivo Rodent Alkaline Comet Assay for the Detection of Genotoxic Carcinogens, Series on Testing and Assessment, Nos. 195 and 196. https://www.oecd.org/env/ehs/testing/Come%20assay%20revised%20pre-validation%20report%202013.pdf.

[58] Burlinson B., Tice R.R., Speit G., Agurell E., Brendler-Schwaab S.Y., Collins A.R., Escobar P., Honma M., Kumaravel T.S., Nakajima M. (2007). In Vivo Comet Assay Workgroup, part of the Fourth International Workgroup on Genotoxicity Testing. Fourth International Workgroup on Genotoxicity testing: Results of the *in vivo* Comet assay workgroup. Mutat. Res..

[59] Paul T., Shukla S.P., Kumar K., Poojary N., Kumar S. (2019). Effect of temperature on triclosan toxicity in *Pangasianodon hypophthalmus* (Sauvage, 1878): Hematology, biochemistry and genotoxicity evaluation. Sci. Total Environ..

[60] Lee J.S., Oh Y., Lee J.S., Kim H.S. (2023). Acute toxicity, oxidative stress, and apoptosis due to short-term triclosan exposure and multi- and transgenerational effects on *in vivo* endpoints, antioxidant defense, and DNA damage response in the freshwater water flea *Daphnia magna*. Sci. Total Environ..

[61] Wang F., Xu R., Zheng F., Liu H. (2018). Effects of triclosan on acute toxicity, genetic toxicity and oxidative stress in goldfish (*Carassius auratus*). Exp. Anim..

[62] Xu X., Lu Y., Zhang D., Wang Y., Zhou X., Xu H., Mei Y. (2015). Toxic assessment of Triclosan and Triclocarban on *Artemia salina*. Bull. Environ. Contam. Toxicol..

[63] Ma Y., Chen C., Wang J.B., Cheng J.L., Shen S., Chen X., Huo J.S. (2022). Triclosan-induced oxidative stress injury and apoptosis by regulating the PI3K/Akt/Caspase-3 signaling pathway in human renal glomerular endothelial cells. Biomed. Environ. Sci..

[64] Zhong R., He H., Jin M., Lu Z., Deng Y., Liu C., Shen N., Li J., Wang H., Ying P. (2022). Genome-wide gene-bisphenol A, F and triclosan interaction analyses on urinary oxidative stress markers. Sci. Total Environ..

[65] Adhikari A., Das B.K., Ganguly S., Nag S.K., Sadhukhan D., Raut S.S. (2023). Emerging contaminant triclosan incites endocrine disruption, reproductive impairments and oxidative stress in the commercially important carp, Catla (*Labeo catla*): An insight through molecular, histopathological and bioinformatic approach. Comp. Biochem. Physiol. C Toxicol. Pharmacol..

[66] Cui Z., He F., Li X., Li Y., Huo C., Wang H., Qi Y., Tian G., Zong W., Liu R. (2023). Response pathways of superoxide dismutase and catalase under the regulation of triclocarban-triggered oxidative stress in *Eisenia foetida*: Comprehensive mechanism analysis based on cytotoxicity and binding model. Sci. Total Environ..

[67] Alfhili M.A., Lee M.H. (2019). Triclosan: An Update on Biochemical and Molecular Mechanisms. Oxid. Med. Cell Longev..

[68] Lee J.S., Oh Y., Park H.E., Lee J.S., Kim H.S. (2023). Synergistic toxic mechanisms of microplastics and triclosan via multixenobiotic resistance (MXR) inhibition-mediated autophagy in the freshwater water flea *Daphnia magna*. Sci. Total Environ..

[69] Pashaei R., Dzingelevičienė R., Putna-Nimane I., Overlinge D., Błaszczyk A., Walker T.R. (2023). Acute toxicity of triclosan, caffeine, nanoplastics, microplastics, and their mixtures on *Daphnia magna*. Mar. Pollut. Bull..

[70] Qu H., Barrett H., Wang B., Han J., Wang F., Gong W., Wu J., Wang W., Yu G. (2021). Co-occurrence of antiseptic triclocarban and chiral anti-inflammatory ibuprofen in environment: Association between biological effect in sediment and risk to human health. J. Hazard. Mater..

[71] Zhang H., Sanidad K.Z., Zhang J., Wang G., Zhang R., Hu C., Lin Y., Haggerty T.D., Parsonnet J., Zheng Y. (2023). Microbiota-mediated reactivation of triclosan oxidative metabolites in colon tissues. J. Hazard. Mater..

[72] Oliver M., Kudłak B., Wieczerzak M., Reis S., Lima S.A.C., Segundo M.A., Miró M. (2020). Ecotoxicological equilibria of triclosan in Microtox, XenoScreen YES/YAS, Caco2, HEPG2 and liposomal systems are affected by the occurrence of other pharmaceutical and personal care emerging contaminants. Sci. Total Environ..

[73] Kenda M., Kuželički N.K., Iida M., Kojima H., Dolenc M.S. (2020). Triclocarban, Triclosan, Bromochlorophene, Chlorophene, and Climbazole effects on nuclear receptors: An *in silico* and *in vitro* study. Environ. Health Perspect..

[74] Pujol E., Blanco-Cabra N., Julián E., Leiva R., Torrents E., Vázquez S. (2018). Pentafluorosulfanyl-containing triclocarban analogs with potent antimicrobial activity. Molecules.

[75] Sreevidya V.S., Lenz K.A., Svoboda K.R., Ma H. (2018). Benzalkonium chloride, benzethonium chloride, and chloroxylenol—Three replacement antimicrobials are more toxic than triclosan and triclocarban in two model organisms. Environ. Pollut..

